# The Legacy Effect in the Prevention of Cardiovascular Disease

**DOI:** 10.3390/nu12113227

**Published:** 2020-10-22

**Authors:** Esther Viñas Esmel, José Naval Álvarez, Emilio Sacanella Meseguer

**Affiliations:** 1 Department of Internal Medicine, Hospital Clínic, Institut d’Investigació Biomèdica August Pi i Sunyer (IDIBAPS), University of Barcelona, Villarroel 170, 08036 Barcelona, Spain; evinas@clinic.cat (E.V.E.); jnaval@clinic.cat (J.N.Á.); 2 Ciber Fisiopatología de la Obesidad y la Nutrición (CIBEROBN), Instituto de Salud Carlos III, 28029 Madrid, Spain

**Keywords:** legacy effect, metabolic memory, cardiovascular disease, diet, diabetes, hypertension, dyslipidaemia

## Abstract

The “legacy effect” describes the long-term benefits that may persist for many years after the end of an intervention period, involving different biological processes. The legacy effect in cardiovascular disease (CVD) prevention has been evaluated by a limited number of studies, mostly based on pharmacological interventions, while few manuscripts on dietary interventions have been published. Most of these studies are focused on intensive treatment regimens, whose main goal is to achieve tight control of one or more cardiovascular risk factors. This review aims to summarise the legacy effect-related results obtained in those studies and to determine the existence of this effect in CVD prevention. There is sufficient data to suggest the existence of a legacy effect after intensive intervention on cardiovascular risk factors; however, this effect is not equivalent for all risk factors and could be influenced by patient characteristics, disease duration, and the type of intervention performed. Currently, available evidence suggests that the legacy effect is greater in subjects with moderately-high cardiovascular risk but without CVD, especially in those patients with recent-onset diabetes. However, preventive treatment for CVD should not be discontinued in high-risk subjects, as the level of existing evidence on the legacy effect is low to moderate.

## 1. Introduction

Cardiovascular disease (CVD) has emerged as a major cause of morbidity and mortality, accounting for 30% of all deaths worldwide. The intensive management of cardiovascular risk factors is required to reduce its incidence, and some authors suggest that achieving tight control at early stages of the disease, before vascular damage has developed, is a determinant of outcomes [1].

The legacy effect concept refers to long-term sustained benefits after a period of intensive treatment intervention, even after cessation of the intervention [2]. Initially described in diabetic patients, it has also been observed in patients with hypertension or hypercholesterolemia [3]. Moreover, the concept of metabolic memory, mostly described in the study of diabetic models, refers to DNA’s ability to store information related to prior poor metabolic control; for example, persistent adverse effects of hyperglycaemia may reduce the potential benefit of subsequent improvements in glucose control, and induce the development of vascular complications in target organs [4,5]. Therefore, achieving good glycaemic control in the early stages of diabetes could be critical in preventing late-stage complications [6,7]. 

Metabolic memory was first described in 1987 when Engerman et al. observed a higher incidence of diabetic retinopathy among dogs in which good glycaemic control was preceded by a longer period of abnormal glucose levels [8]. Likewise, long-term overproduction of fibronectin, both by endothelial cells cultured in a high-glucose medium and by the kidneys of streptozocin-induced diabetic rats after the restoration of near-normoglycemia, was also detected [9]. Further investigation in 1993 showed that transplanting the islets of Langerhans shortly after diabetes mellitus onset in rats could reverse diabetic retinopathy [10].

However, most studies examining the legacy effect on CVD and its related risk factors for vascular complications are based on pharmacological interventions, with low emphasis on dietary interventions; thus, the long-term effects of strict dietary regimens remains unknown. In this review, we focus on the recent evidence of the legacy effect and metabolic memory in CVD development after intensive pharmacological and non-pharmacological interventions addressing cardiovascular risk factors.

## 2. Methods

The aim of this narrative review was to assess those scientific studies that evaluated the existence of the legacy effect in the prevention of CVD after a nutritional or pharmacologic intervention. We searched for scientific studies published in the last ten years and written in English in PubMed Medline Database by using specific search terms (“legacy effect”, “metabolic memory”, “cardiovascular disease”, “diet”, “diabetes”, “hypertension” and “dyslipidemia”). In addition, recent reviews and meta-analysis about the legacy effect, metabolic memory and its pathophysiological mechanisms were also included. To sensitise the search and select the best articles, we used the aforementioned keywords and applied different Boolean operators. Finally, a total of 64 articles were selected for this review. Most of them were clinical trials in humans, and there was also a small proportion performed in animal models. The differences observed among the outcomes of the trials and their post-trial follow-up studies have been described by using the difference in means, risk ratio (RR), odds ratio (OR) and hazard ratio (HR) measures. The publication, attrition and co-intervention bias, and the baseline characteristics of the patients included in the trials should be taken into consideration for the interpretation of the results.

## 3. Pathophysiological Mechanisms Involved in Metabolic Memory

Multiple mechanisms have been described as relevant to the development of the legacy effect. Diabetic models have been used to thoroughly study the concept of metabolic memory. Furthermore, studies performed on hypertension models have shown that similar mechanisms are involved in the maintenance of beneficial post-intervention effects.

### 3.1. Oxidative Stress

**Figure 1:**
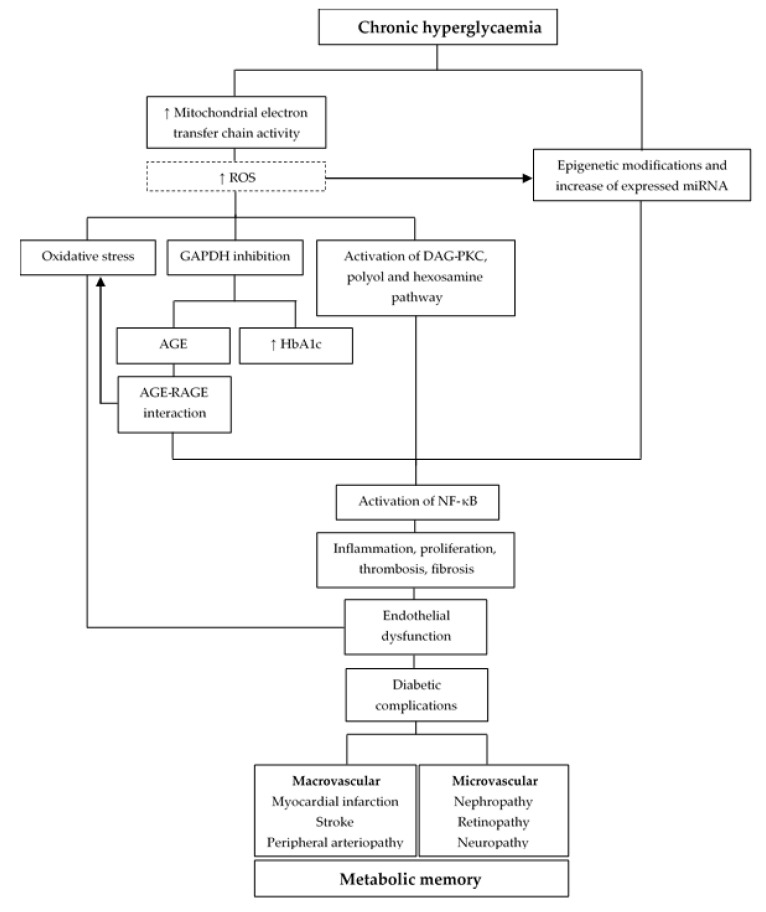
Pathophysiological mechanisms of metabolic memory in chronic hyperglycaemic conditions. Abbreviations: AGE: advanced glycation end-products; DAG-PKC: diacylglycerol-protein kinase C; GAPDH: glyceraldehyde 3-phosphate dehydrogenase; HbA1c: glycated haemoglobin; miRNA: micro RNA; NF-κB: nuclear factor κB; RAGE: AGE receptor; ROS: reactive oxygen species.

Chronic hyperglycaemia induces uncoupling of the mitochondrial electron transfer chain in endothelial cells, causing excessive production of superoxide anion and other reactive oxygen species (ROS), thereby increasing cellular oxidative stress and inducing endothelial dysfunction. Moreover, ROS can cross membranes and damage macromolecules (nucleic acids, mitochondrial DNA and proteins), many of which may have longer half-lives, and hence may exert metabolic effects. These oxidative stress markers can persist in endothelial cells despite glucose normalisation after prolonged hyperglycaemia, suggesting a metabolic memory phenomenon (Figure 1) [4,5]. Furthermore, postprandial glucose oscillations in diabetic patients have been associated with increased ROS marker levels and vascular stress; the cells’ inability to adapt to this dynamic environment leads to cell damage and elevated collagen and fibronectin levels that remain abnormal for several days after glucose levels have normalised [11,12,13]. In hypertension models, the overproduction of ROS by NADPH oxidase upregulation due to renin-angiotensin system activation appeared to persist after cessation of the hypertensive period and was related to deleterious effects. Otherwise, the intensive blocking of the renin-angiotensin system showed a long-term reduction in the overproduction of ROS [14]. 

### 3.2. Non-Enzymatic Glycosylation of Proteins and Chronic Inflammation

Superoxide anion inhibits glyceraldehyde 3-phosphate dehydrogenase (GAPDH) activity, leading to the accumulation of all glycolytic intermediates, which can react non-enzymatically with proteins, lipids, and nucleic acids to form advanced glycation end-products (AGEs). AGEs may accelerate ageing processes, and they cannot be degraded easily by the enzymes involved in normal metabolism. In addition, the most exposed proteins, such as haemoglobin, are also highly glycated [4]. AGEs also promote oxidative stress, vascular hyperpermeability, pathological angiogenesis, and thrombogenic reactions via interaction with their receptors (RAGEs), which promote endothelial dysfunction and atherosclerosis [5,15,16]. Hyperglycaemia also causes the activation of the diacylglycerol (DAG)-protein kinase C (PKC) pathway, fructose production, and increased flux through the hexosamine pathway, which intensifies the expression of various adhesive molecules, proinflammatory cytokines, and growth factors. These mechanisms activate nuclear factor κB (NF-κB), a rapid-response transcription factor involved in inflammatory reactions and proapoptotic programs in diabetes. 

Even after glycaemic control has been achieved, AGEs may be used as markers to measure cumulative diabetic exposure, especially in those with vascular complications, whereas intensive treatment is associated with significantly lower AGE levels. These pathways are responsible for metabolic memory and contribute to the development and progression of CVD and diabetic complications. Glycaemic memory can be measured by glycated haemoglobin (HbA1c), although AGEs have also been correlated with retinopathy progression, independent of HbA1c level [16]. Some medications could alter these pathways and, consequently, prevent the development of CVD. Metformin, pioglitazone, glucagon-like peptide-1 (GLP-1) receptor agonists and dipeptidyl peptidase-4 (DPP-4) inhibitors have been shown to prevent AGE formation or decrease inflammation by blocking AGE and NF-κB pathways, reversing the metabolic memory phenomenon [12,16,17,18]. Also, angiotensin-converting-enzyme inhibitors (ACEI) and angiotensin II receptor type 1 (AT-1) blockers can reduce AGE formation and, subsequently, the inhibition of superoxide generation [17]. It is widely recognised that benefits of renin-angiotensin-aldosterone system (RAAS) inhibition extend beyond blood pressure (BP) reduction and may last even after switching from an intensive BP-lowering strategy to a conventional one; its effectiveness in preventing diabetic complications indirectly suggests that RAAS dysregulation is involved in triggering organ damage in diabetic patients [19].

### 3.3. Epigenetic Modifications

Chronic hyperglycaemia can induce epigenetic modifications, which could underlie endothelial dysfunction and the development of metabolic memory in diabetic complications. Recent research suggests that DNA methylation and post-translational histone modifications in a hyperglycaemic environment could lead to enhanced expression of proinflammatory genes, such as the increased expression of the key p65 subunit of NF-κB in various target tissues, which persists long after restoration of normoglycemia. Moreover, in cell and animal models exposed to transient-high and subsequent normal glucose levels, an increase in differentially expressed miRNAs has been observed; these may play a role as regulators of endothelial dysfunction in metabolic memory by increasing the constitutive activation of NF-κB pathway. However, the potential reversal of glucotoxicity and lipotoxicity depends on how long the patient has been exposed to this poor metabolic environment [20,21].

## 4. Legacy Effect after Dietary Intervention 

Few studies have assessed the legacy effect after dietary intervention. Most of these have been done in animals. Below, we describe the most relevant findings.

One study showed that mice switched from dietary restriction (DR) to ad libitum (AL) intake had significantly better glucose tolerance at 6–10 months compared to the AL group, suggesting that there was positive glycaemic memory in the DR group [22,23,24]. Moreover, it was also observed that short-term reversion to a normal diet in rats after initial exposure to a high-fat diet (HFD) could not restore the insulin resistance-induced complications. However, administering metformin to these animals induced remarkable amelioration of anomalies associated with insulin resistance and endothelial dysfunction via lipotoxicity reduction [25,26]. In another study, mice fed a HFD (60% kcal from fat) for 21 weeks were compared to those fed a low-fat diet (LFD, 10% kcal from fat); the HFD animals developed type 2 diabetes mellitus (T2DM) and gained much more weight compared to mice fed an LFD. During the 4-week intervention period following diabetes onset, insulin-treated mice maintained on an HFD exhibited significantly improved glucose tolerance test results compared to sham-treated animals. However, mice remaining on a HFD following cessation of the insulin treatment exhibited no benefit from the early insulin therapy and continued to gain weight, had worse glucose tolerance test results, and displayed significantly higher fasting insulin, C-peptide, and leptin levels compared to sham-untreated controls, and early insulin therapy in mice maintained on the LFD. In fact, early insulin therapy only conferred a beneficial effect in animals switched to an LFD after the insulin treatment. These findings indicated that the legacy effect of early insulin-treatment was only observed in diabetic mice switched to an LFD after the insulin treatment and emphasised the vital role of diet adherence in diabetes control at any stage of disease progression [27].

The legacy effect was also confirmed in rats subjected to an HFD, when time-restricted feeding was alternated with AL feeding, with no significant differences in body weight observed between the alternated feeding and the control group (AL feeding only) in a short term-study (12 weeks). However, in the long-term study (25 weeks), when mice were maintained on time-restricted feeding for 13 weeks and then switched to AL feeding for 12 weeks, they showed significantly less body weight gain after returning to an HFD compared to the control group, which were maintained on time-restricted feeding throughout the 25 weeks (112% versus 51% body weight increase, respectively) [28]. Further research performed on male rats showed that maintaining an HFD induced persistent changes in sperm cells that could be transmitted to the female descendants, impairing their glucose tolerance and insulin secretion [29].

To our knowledge, there is limited evidence assessing the existence of a legacy effect in humans after dietary intervention. The effects of caloric restriction (approximately 25% of daily energy intake) or intermittent fasting (up to 18 h/day) in humans have been demonstrated mainly in observational studies, and only one clinical trial, the Comprehensive Assessment of the Long-term Effects of Reducing Energy Intake (CALERIE) study, was performed. Both strategies improve general health indicators and slow or reverse ageing and disease processes, such as obesity, insulin resistance, dyslipidaemia, hypertension and inflammation, although intermittent fasting seems to exert a greater effect. Improvements in health indicators typically begin within the first month, after the start of intermittent fasting, and then dissipate over a period of several weeks after resumption of a normal diet [30]. This effect was also evaluated in a small clinical trial of 24 obese older patients (mean age 65–79 years) after caloric restriction (CR) intervention plus resistance training (RT) compared to RT intervention for five months. Despite clinically meaningful weight loss and concurrent favourable shifts in total body and thigh composition in the CR+RT group compared to RT group during the intervention period, these improvements were generally not sustained over the long-term (18 months). At the end of the study, only weight was significantly lower compared to baseline in the CR+RT group. Therefore, this trial showed a temporary legacy effect which lost intensity during the follow-up [31].

The Oslo cardiovascular study was a five year randomised intervention conducted in healthy middle-aged men at high risk of coronary heart disease (CHD) assigned to a dietary advice group (main objectives: to reduce daily intake of saturated fats, sugar and alcohol; to increase daily intake of fish, vegetables and fruit; to reduce weight; to quit smoking) compared to a control group. At 40 years of follow-up, a significant reduction (19%) in the risk of death at first myocardial infarction (MI) was detected in the intervention group (HR 0.71, 95% confidence interval (CI), 0.51–1.00), with no significant difference in total mortality. The legacy effect was achieved through the dietary intervention (as evidenced by lower cholesterol and serum triglyceride levels and weight reduction) because few men quit smoking; therefore, the effect of this measure was small [32].

In the Look Action for Health in Diabetes (Look-AHEAD) trial, overweight/obese patients with T2DM were randomly assigned to an intensive lifestyle intervention to achieve and maintain a weight loss ≥7%, which included: hypocaloric diet (1200–1800 kcal/day; less than 30% of daily energy intake from fat and less than 10% from saturated fat; at least 15% of daily energy intake from protein), physical activity and diabetes education, compared to standard care for one year. Patients included in the intensive lifestyle intervention had greater weight reduction with better control of cardiovascular risk factors (glycaemic control, systolic BP and lipid profile), and more improvement in fitness levels throughout the four year follow up period compared to the control group [33].

## 5. Legacy Effect in Diabetic Patients after Intensive Glycaemic Control

Several clinical trials have assessed legacy effects in different populations of patients with diabetes, the main results of which are listed below. The specific data from each article are shown in Table 1.

The Diabetes Control and Complications Trial (DCCT) was performed in patients with type 1 diabetes mellitus (T1DM), who were randomly assigned to either intensive or standard glycaemic control regimens. After 6.5 years of intervention, the development of severe proliferative or non-proliferative retinopathy (HR 0.47, 95% CI, 0.14–0.67), clinical neuropathy (HR 0.60, 95% CI, 0.38–0.74), and microalbuminuria (HR 0.39; 95% CI, 21–52) was significantly lower in the intensive treatment group [34]. After the DCCT trial, patients were followed up for 17 years in the Epidemiology of Diabetes Interventions and Complications (EDIC) study. A legacy effect was observed in the former intensive-therapy group during this period, as evidenced by a significant reduction in the prevalence of cardiovascular events (nonfatal MI, stroke, or death from CVD; HR 0.43; 95% CI, 0.12–0.79), and nephropathy (HR 0.54, 95% CI, 0.34–0.84) (Table 1) [35]. 

The United Kingdom Prospective Diabetes Study (UKPDS) recruited patients with newly diagnosed T2DM (six months from diagnosis); after a three-month diet (main features: low and moderately high daily intake of saturated fat and fibre, respectively; about 50% of daily caloric intake from carbohydrates; hypocaloric diet in overweight patients), they were randomised to intensive glucose control with medication (sulfonylurea, insulin, metformin) or to conventional dietary treatment, and they were followed-up for ten years. Intensive glucose management was associated with a significant reduction for any diabetes-related endpoint (12%) and for any diabetes-related death (10%) compared to the control group. Most of the risk reduction in the “any diabetes-related aggregate” endpoint was due to a 25% risk reduction in microvascular endpoints. However, there was no significant reduction in macrovascular endpoints between both arms of the study. Moreover, patients in the intensive group had more hypoglycaemic episodes than those in the conventional one [36]. Although differences between both groups in HbA1c levels were lost after the first year, a clearly different incidence in clinical endpoints in the post-trial follow-up of the UKPDS was observed. Thus, relative reductions in risk persisted at ten years for any diabetes-related endpoint (RR 0.91, CI 95%, 0.83–0.99), for microvascular disease (RR 0.76, CI 95%, 0.64–0.89), MI (RR 0.85, CI 95%, 0.74–0.97), and for death from any cause (RR 0.87, CI 95%, 0.79–0.96), and emerged over time in the intensive therapy group, as more events occurred. In the metformin group (overweight patients), significant risk reductions persisted for any diabetes-related endpoint (RR 0.79, CI 95%, 0.66–0.95), MI (RR 0.67, CI 95%, 0.51–0.89), and death from any cause (RR 0.73, CI 95%, 0.59–0.89) (Table 1) [37,38]. 

The Action to Control Cardiovascular Risk in Diabetes (ACCORD) trial included patients with T2DM with a mean duration of ten years and previous cardiovascular events or multiple cardiovascular risk factors. The patients were assigned to receive intensive or standard therapy (targeting HbA1c <6% versus vs. <7.9%, respectively) for 3.7 years, with a combination of different hypoglycaemic drugs including insulin [39]. It was not observed statistically significant benefit during the intervention period nor after ending the trial in the intensive treatment group, except for the rate of nonfatal MI, which was lower than in the standard therapy group (HR 0.76, 95% CI, 0.62–0.92). In fact, a significant increase in cardiovascular mortality (HR 1.35, 95% CI, 1.04–1.76), and death from any cause (HR 1.22, 95% CI, 1.01–1.46) was detected during and after the ACCORD trial in the intensive treatment group, attributed to a higher incidence of severe hypoglycaemia in those patients (Table 1) [39,40]. 

The Veterans Affairs Diabetes Trial (VADT) was performed in patients with poorly controlled long-standing T2DM (mean duration of 11.5 years), 40% of whom had already suffered a cardiovascular event. They were randomly assigned to intensive or standard therapy for a median of 5.6 years. Other cardiovascular risk factors were treated uniformly. The intensive glucose-lowering treatment led to a between-group difference of 1.5 percentage points in HbA1c levels (6.9% in the intensive-therapy group vs. 8.4% in the standard-therapy group) during the in-trial period. The only benefit observed was a slower progression of microalbuminuria in the intensive treatment group. However, a significantly lower incidence of CVD (HR 0.83, 95% CI, 0.70–0.99) in patients originally assigned to intensive therapy was observed after ten years of follow-up. Nevertheless, over a 15-year follow-up period, the risks of major cardiovascular events (HR 0.91, 95% CI, 0.78–1.06), or death from any cause (HR 1.02, 95% CI, 0.88–1.18) were not lower in the intensive-therapy group than in the standard therapy group, suggesting a modest and transient long-term cardiovascular benefit of intensive glucose-lowering therapy in patients with more advanced diabetes (Table 1). In fact, the cardiovascular benefit (legacy effect) of intensive therapy lasted when HbA1c curves of both groups were separated (Table 1) [41,42,43].

The Anglo-Danish-Dutch study of Intensive Treatment in People with Screen-Detected Diabetes in Primary Care (ADDITION) study recruited middle-aged adults with a moderate to high risk of T2DM for diabetes screening, in order to evaluate the risk of CVD and mortality among incident cases of T2DM in a screened group compared to an unscreened population. Participants diagnosed with T2DM were offered intensive multifactorial treatment and compared to those receiving routine care for five years, with a reduction in fatal and nonfatal CVD of 17% shown. After a 10-year follow-up, patients diagnosed after population-based screening had a significant 21% lower risk in all-cause mortality (HR 0.79, 95% CI, 0.74–0.84) and a significant 16% lower risk in cardiovascular events (HR 0.84; 95% CI, 0.80–0.89), but no significant risk reduction on cardiovascular mortality compared to unscreened patients [44]. Moreover, population-based diabetes screening was associated with less need for insulin therapy after ten years and slightly better long-term glycaemic control compared to patients diagnosed during usual care (Table 1) [45].

The Diabetes and Aging Study was a large observational study of newly diagnosed T2DM patients and followed those with long survival post-diagnosis (≥10 years). The study assessed the impact of HbA1c levels ≥6.5% in the first year after T2DM diagnosis on later CVD risk. The risk of micro- and macrovascular events was higher in those patients with HbA1c levels ≥6.5% compared to those with lower levels, whereas mortality risk was significantly higher in patients with HbA1c levels ≥8%. These results suggest it is necessary to achieve normoglycemia in the first 12 months after T2DM diagnosis to generate a legacy effect (Table 1) [46].

## 6. Legacy Effect after Blood Pressure Control 

Several clinical trials have assessed legacy effects in different populations of hypertensive patients, the main results of which are listed below. The specific data from each article are shown in Table 2.

A separate UKPDS post-trial follow-up study assessed whether risk reductions for micro- and macrovascular complications were achieved and maintained in a subgroup of hypertensive subjects with newly diagnosed T2DM with tight versus less-tight BP control with captopril or atenolol. However, differences in BP between groups (tight vs. less tight) disappeared within two years after trial termination, and most cardiovascular benefits were not sustained during the 10-year post-trial follow-up period. Only a reduced risk for peripheral vascular disease associated with tight BP control was significant (RR 0.50, 95% CI, 0.28–0.92). Hence it would be necessary to maintain good BP levels over time to preserve this previous benefit (Table 2) [36,47].

The Systolic Hypertension in the Elderly Program (SHEP) trial was performed in elderly patients with isolated systolic hypertension who were randomised to chlortalidone therapy or placebo (plus atenolol or matching placebo, if BP remained uncontrolled). Over a mean follow-up of 4.5 years therapy, reduction in fatal or nonfatal stroke (RR 0.64, 95% CI, 0.50–0.82), MI (RR 0.67, 95% CI, 0.47–0.96), and heart failure (RR 0.51, 95% CI, 0.37–0.71) in the chlortalidone group compared to placebo was observed. However, there was no significant risk reduction in all-cause and cardiovascular mortality. After a 22-year follow-up, significant, albeit modest life expectancy gains free from CVD-related deaths were observed in the chlortalidone group, corresponding with approximately one day (HR 0.89, 95% CI, 0.80–0.99) gained for each month of treatment. (Table 2) [48].

In the Antihypertensive and Lipid Lowering Treatment to Prevent Heart Attack Trial (ALLHAT), 32,804 hypertensive patients with at least another cardiovascular risk factor were randomised to receive chlorthalidone, amlodipine, or lisinopril for 4 to 8 years, and thereafter, passive surveillance continued for a total follow-up of 8 to 13 years. During the in-trial period, no statistically significant differences in CHD or nonfatal MI were observed. In the post-trial follow-up, the only significant differences were observed in secondary outcomes, such as heart failure and stroke mortality, which were higher with amlodipine and lisinopril, respectively, compared to chlorthalidone. However, a significant treatment-by-race interaction was detected and, after accounting for multiple comparisons, none of these results were deemed significant (Table 2) [49].

The Randomised Olmesartan And Diabetes MicroAlbuminuria Prevention (ROADMAP) trial included patients with T2DM with at least one additional cardiovascular risk factor and normoalbuminuria. They were randomly assigned to olmesartan or placebo for 3.2 years; the main outcome was significantly delayed microalbuminuria onset in the olmesartan group. After a 6.5-year observational follow-up period, researchers concluded that congestive heart failure (CHF) requiring hospitalisation (OR 0.23, CI 0.06–0.85) and diabetic retinopathy (OR 0.34, CI 0.15–0.78) were significantly lower in the former olmesartan group, indicating a legacy effect in these outcomes (Table 2) [50,51].

The Second Australian National BP study (ANBP2) observed a lower risk of cardiovascular (HR 0.47, 95% CI, 0.27–0.81) and all-cause mortality (HR 0.63, 95% CI, 0.46–0.86) in the “treatment-naive” group (participants without BP-lowering medication at study registration) compared to BP-lowering medication or “previous treatment” group (BP-lowering treatment at registration; median duration of previous therapy was five years) during the in-trial period in an elderly cohort. No differences were found between groups with respect to randomised treatment allocation to either ACEI or diuretic-based regimens; likewise, there were no differences in cardiovascular outcomes or all-cause mortality when the data from the in-trial and ten-year post-trial follow-up were combined (Table 2) [52].

The Anglo-Scandinavian Cardiovascular Outcomes Trial (ASCOT) enrolled patients with hypertension and at least three other cardiovascular risk factors. In the BP-lowering arm, patients were randomised to either an amlodipine-based regimen (adding perindopril as required) or an atenolol-based regimen (adding bendroflumethiazide and potassium as required). The in-trial results demonstrated a significant reduction in cardiovascular events, mortality and all-cause mortality in subjects assigned to the amlodipine-based group, but no overall difference in cardiovascular mortality (HR 0.90, 95% CI, 0.81–1.01) among treatments. These results could suggest that not all hypotensive drugs are equally effective in preventing CVD, although the resulting BPs are similar. After 16 years of total follow-up (ASCOT Legacy Study), there was no overall difference in all-cause mortality between treatments, although significantly fewer deaths from stroke (HR 0.71, 95% CI, 0.53–0.97) occurred in the amlodipine-based treatment group (Table 2) [53].

Finally, a systematic review and meta-analysis of three clinical trials were performed to analyse whether early versus late initiation of antihypertensive treatment was better at reducing cardiovascular morbidity and mortality. The trials involved 4746 mildly hypertensive (systolic BP 140–159 mmHg) middle-aged patients free of CVD at baseline and with low cardiovascular risk. No differences were seen between strategies during the in-trial period (5 years) or during post-trial follow-up (ten years). Therefore, the review showed no clinically adverse legacy effect on mortality or major CVD with delayed pharmacotherapy in middle-aged mildly-hypertensive subjects with low cardiovascular risk [54].

## 7. Legacy Effect after Lipid Control 

Several clinical trials have assessed the legacy effects in different populations of patients with dyslipidaemia the main results of which are listed below. The specific data from each study are shown in Table 3.

The ALLHAT lipid-lowering trial (LLT) evaluated the impact of large, sustained cholesterol reductions in all-cause mortality in a cohort of hypertensive patients with at least one other cardiovascular risk factor. All-cause mortality did not differ significantly between the pravastatin and usual care treatment groups (RR 0.99, 95% CI, 0.89–1.11) at six years of follow-up. Similarly, no significant reductions in CHD events (RR 0.91, 95% CI, 0.79–1.04) were observed at the end of the follow-up period (Table 3) [55].

The West of Scotland Coronary Prevention Study (WOSCOPS) was a primary prevention trial performed in men (45 to 65 years old) with hypercholesterolemia who were randomised to pravastatin or placebo for five years. The 20-year follow-up study after the initial intervention identified a legacy benefit in the pravastatin group compared to placebo, with a significant reduction in all-cause mortality (HR 0.87, 95% CI, 0.80–0.94), attributable mainly to a 21% decrease in cardiovascular death (HR 0.79, 95% CI, 0.69–0.90), reduction in hospital admissions for any coronary event (18%), for MI (24%), and for heart failure (35%). The authors suggested that these benefits may be the result of reduced infarct size, prevention and regression of atherosclerotic changes, or pleiotropic effects of statins. On the other hand, there was no difference in non-cardiovascular or cancer death rates between groups (Table 3) [56]. 

The ASCOT lipid-lowering arm (LLA) is a substudy of the ASCOT study previously reported. ASCOT-LLA enrolled ASCOT study participants who had total cholesterol of 6.5 mmol/L or less and no previous lipid-lowering treatment. They were randomised to receive atorvastatin or placebo as primary prevention for CHD; lower nonfatal MI and fatal CHD was demonstrated in the atorvastatin group during the interventional period of the study. The legacy effect was studied for 16 years after initial randomisation and also showed a significant risk reduction in cardiovascular mortality (HR 0.85, 95% CI, 0.72–0.99) in those assigned to atorvastatin group. No differences in all-cause mortality or stroke were seen between the atorvastatin and placebo groups during the post-trial study period (Table 3) [52].

The ACCORD-Lipid study included a subgroup of ACCORD study participants. Briefly, T2DM patients were randomised to receive simvastatin plus fenofibrate vs. simvastatin plus placebo for five years; no evidence of a beneficial effect of statin-fibrate combined treatment compared to statins alone on cardiovascular outcomes and mortality was found. However, a beneficial reduction in major CHD (HR 0.65, 95% CI, 0.48–0.90) was observed in a subgroup of participants with dyslipidaemia. The extended post-trial follow-up study (ACCORDION) showed lower rates of all-cause mortality, cardiovascular mortality, nonfatal MI, CHF and major CHD in the simvastatin-fibrate group in the ten years following randomisation. Moreover, the trial period’s combined statin-fibrate treatment arm conferred a beneficial legacy effect, which was observed in the post-trial follow-up on all-cause mortality (HR 0.65, 95% CI, 0.45–0.94). Fibrate therapy, offered as an add-on to statin therapy, may be beneficial for people with diabetes with hypertriglyceridemia and/or reduced HDL-C (Table 3) [57]. 

A recent meta-analysis, including eight placeboes vs. statin randomised clinical trials for primary and secondary cardiovascular prevention, evaluated the legacy effect during a mean post-trial follow-up ranging from 1.6 to 15.1 years. The results mostly showed a significant reduction in cardiovascular and all-cause mortality within the trial period, and less benefit in the post-trial period. The legacy effect was observed in relation to lower all-cause mortality but, appeared to have no effect on CVD mortality. Additionally, in a subgroup analysis, there appeared to be a greater legacy effect when statins were used for primary prevention compared to secondary prevention in CVD and all-cause mortality (HR 0.87 and 0.90, respectively), suggesting the importance of long-term prevention in these patients [58].

Another meta-analysis carried out by Hirakawa et al. (62) analysed the legacy effect in the post-trial follow up (mean duration six years) after an intervention period with antihypertensive and lipid-lowering treatments. The study demonstrated significant reductions in all-cause and cardiovascular mortality (about 9% and 12%, respectively) after discontinuation of antihypertensive and lipid-lowering treatment during the overall follow-up, although this effect was lower than that observed during the in-trial phase. Furthermore, progressive attenuation of post-trial benefits was noted as the length of the post-trial observation increased. However, no clear differences between the effects of BP or lipid-lowering therapies were detected across trials studying different types of therapies or patient populations. These findings indicate that it is important to continue antihypertensive and lipid-lowering treatment in the long-term to provide optimal cardiovascular protection [59].

## 8. Legacy Effect after Multifactorial Intervention

The Action in Diabetes and Vascular Disease (ADVANCE) trial was performed in 11,140 T2DM patients ≥55 years old, with at least one additional risk factor for CVD. The trial proposed a double strategy to optimise glycaemic control (intensive glucose control or standard control) and BP control (perindopril-indapamide or placebo) for five years. During the intervention phase, there was a reduction in microvascular events (14%), primarily due to reduction in nephropathy incidence in the intensive glucose-control group, as well as a reduction in the relative risk of all-cause mortality (14%) and cardiovascular mortality (18%) in the BP control group. The six-year post-trial follow-up study found a significant reduction in all-cause mortality (HR 0.91, 95% CI, 0.84–0.99), and cardiovascular mortality (HR 0.88, 95% CI 0.77–0.99) in those patients originally treated with perindopril-indapamide compared to placebo. Although the confidence limits were wide, the results suggested that one death from any cause would be prevented for every 79 patients assigned to active therapy for five years. On the other hand, there was no evidence that the effects of the treatment could be inferred from initial BP levels or concomitant use of other treatments at baseline. However, intensive glucose control did not provide any long-term benefits during the intervention period nor after post-trial follow-up (Table 4) [60,61,62].

The Steno-2 study recruited T2DM patients (*n* = 160; mean age 55 years) with microalbuminuria who were randomly assigned to standard treatment or intensified multifactorial intervention during a mean follow-up of 7.8 years. This intervention included simultaneous control of glucose levels, BP, lipid profile, and antiplatelet therapy through pharmacological and non-pharmacological intervention. The latter included dietary advice (total daily intake of fat ≤30% of total daily energy intake and less than 10% kcals as saturated fat), light to moderate exercise at least 30 min three to five times a week, and smoking cessation. During the active intervention period, there was risk reduction for nephropathy progression (OR 0.27, 95% CI, 0.10–0.75), retinopathy progression (OR 0.45; 95% CI, 0.21–0.95), and autonomic neuropathy progression (OR 0.32, 95% CI, 0.12–0.78), as well as for cardiovascular complications and all-cause mortality combined (OR 0.45, 95% CI, 0.22–0.93) in the intensive treatment group. After a 21-year follow-up from randomisation, a significant reduction in all-cause mortality (45%), cardiovascular mortality (62%), macroalbuminuria (48%), retinopathy progression (33%), and autonomic neuropathy (41%) was demonstrated in the intensive treatment arm, suggesting a legacy effect from intensive multifactorial treatment compared to the standard approach. For patients in the intensive treatment group, death and time to first cardiovascular event was delayed by 7.9 and 8 years, respectively, compared to patients in the standard treatment group (Table 4) [63,64].

## 9. Discussion

Most studies evaluating the legacy effect on the primary or secondary prevention of CVD are clinical trials promoted and financed by the pharmaceutical industry in which an observational study has continued at the end of the intervention phase. As a consequence, the legacy effect has been assessed mainly after pharmacological intervention. Few studies have been carried out after a nutritional intervention, and most focused on animal and diabetic models. 

Different clinical outcomes have been evaluated, including microvascular complications (retinopathy, neuropathy, nephropathy), major cardiovascular events (heart failure, MI, stroke, and ischemic gangrene) and mortality, as well as analytical parameters. The results obtained have been quite heterogeneous, probably due to differences in the baseline characteristics of the patients included, the type and duration of the interventions, and post-trial follow-up. In general terms, the legacy effect has been observed to be less intense than that achieved in the intervention phase and tends to diminish with longer follow-up. All the data suggest that this effect is greater and longer lasting in patients without known CVD undergoing intensive therapy for recent-onset diabetes, hypertension or dyslipidaemia.

A limited number of studies related to the legacy effect after nutritional intervention have been published to date. In studies performed with rats and mice, some authors have demonstrated that after CR (reduction of daily caloric intake by approximately 25%) or intermittent fasting, glycaemic control and insulin resistance could improve for several weeks. The evidence in humans is lacking, as health benefits of CR or intermittent fasting have been evaluated mainly in observational studies and a single clinical trial (CALERIE Study). The effect during the intervention period appears to be favourable but disappears shortly after the intervention ends. Therefore, CR or intermittent fasting has not been shown to generate a legacy effect in humans [30]. In addition, we would like to highlight safety concerns regarding intermittent fasting in patients with diabetes mellitus due to the higher risk of hypoglycemia. Data from the Oslo Cardiovascular study suggest that systematic advice on a healthy diet and smoking cessation for five years could be associated with a legacy effect translated into a reduced risk of cardiovascular mortality in the next 40 years. However, it is difficult to ensure that no confounding factors could have altered these results [32]. Currently, there are no data available on the possible legacy effect in other, more recent nutritional intervention studies, such as the PREDIMED (Prevención con Dieta Mediterránea) Study [65]. The limited scientific evidence published on the possible legacy effect in CVD prevention after a dietary intervention may be due to multiple factors, including: difficulty in maintaining high adherence to the proposed nutritional intervention, absence of specific biomarkers of dietary compliance and difficulty in having a real control group or blinding the interventions. In addition, these studies require a very long follow-up as well as a high economic cost that can be more difficult to finance if there is no financial support from public institutions. Due to all these limitations, there are few nutritional intervention studies that have carried out a long follow-up at the end of the intervention [66,67].

The first scientific evidence of the legacy effect in patients with diabetes comes from the DCCT and the UKPDS studies, in which an intensive glycaemic control compared to standard treatment resulted in a significant reduction in cardiovascular mortality, nonfatal MI, stroke and nephropathy. These beneficial effects persisted for more than a decade after the intervention was finished [35,37]. Furthermore, the VADT trial provided the first evidence of the legacy effect in the reduction of a composite of major cardiovascular events (MI, stroke, CHF or amputation for ischemic gangrene) in patients with poorly controlled and long duration T2DM after ten years of randomisation. Notwithstanding, these significant cardiovascular outcomes disappeared over a longer follow-up period (15 years) [42,43]. On the other hand, the ADVANCE and ACCORD trials, which included long-standing T2DM patients, showed no legacy effect in CVD. Differences observed among these trials may be explained due to the fact that the DCCT and UKPDS studies recruited younger patients with new-onset diabetes without known CVD, in contrast to the characteristics of the patients included in the ADVANCE and ACCORD studies [40,62].

Several clinical trials have analysed the legacy effect after tight BP control with controversial results. On the one hand, the SHEP and the ROADMAP trials showed reductions in cardiovascular mortality, retinopathy and delayed onset of microalbuminuria, whereas the ASCOT study only demonstrated a small reduction in stroke mortality in the amlodipine-treatment group at the end of the entire follow-up [49,50,52]. On the contrary, the HDS and the ALLHAT studies did not provide clear evidence of a legacy effect, suggesting that this beneficial effect in patients with higher cardiovascular risk and probable subclinical CVD is unlikely to be achieved once in the post-trial phase, when BP is less strictly controlled. In addition, the benefits of antihypertensive treatment on major cardiovascular outcomes usually appear shortly after treatment implementation, and are attenuated when BP differences between groups are lost [47,48]. Therefore, based on clinical trials conducted, it appears that the legacy effect does not exist or seems to be mild and transient after tight antihypertensive regimens.

Different lipid-lowering trials, including ASCOT, WOSCOPS and ACCORD lipid studies, have also observed a legacy effect, resulting in a reduction in all-cause mortality and CHD mortality for 10–20 years after ending the interventional phase (statin or fibrates treatment), whereas the ALLHAT-LLT study did not provide evidence of a legacy effect after intensive lipid-lowering treatment [52,55,56,57]. It should be noted that in those trials in which a legacy effect was observed, the statin, a drug with pleiotropic effects, was compared against placebo. Moreover, the observation that five years of statin therapy led to a lower long-term risk of all-cause and CVD mortality raises the question of whether treatment with statins for 5–10 years would be sufficiently beneficial, while limiting lifetime exposure to the drug. However, there are still concerns about whether this therapeutic strategy could be effective in any patient, or in select populations only.

Until now, the legacy effect after multifactorial intervention has only been observed in the Steno-2 study, which included pharmacological and non-pharmacological measures. Indeed, lower mortality, CVD incidence and microvascular complications were detected in the intensive treatment arm of the trial compared to the standard treatment group. Surprisingly, the results were obtained in a sample of only 160 patients with intermediate cardiovascular risk but without CVD at baseline. It has been suggested that the positive long-term cardiovascular effects must be the result of a synergistic effect of the multifactorial intervention on cardiovascular risk factors [64]. Although these results are impressive, they have not been reproduced in subsequent studies.

The knowledge of pathophysiological mechanisms underlying the legacy effect adds plausibility to its existence. The best-known mechanisms are those related to the deleterious effects of hyperglycaemia through the development of metabolic memory induced during the first years of diabetes onset, which cannot be reversed with better glycaemic control. Thus, early interventions against hyperglycaemia could reduce ROS production and oxidative stress in the mitochondria of endothelial cells, decrease AGE formation and RAGE expression, and therefore, prevent activation of inflammatory processes and epigenetic changes in the arterial walls in the long term [4,5,15,16,17,20,21]. Likewise, dysregulation of RAAS may be involved in vascular complication development, through increased oxidative stress and AGE formation. However, these mechanisms are not yet fully understood [19].

This review has several strengths and limitations. Firstly, one strength involves the inclusion of well-designed multicentre prospective clinical trials with large participant samples during the interventional phase. Secondly, most trials have a long post-interventional follow-up (6–20 years), which is enough time to detect differences between groups. Likewise, the study of different types of cardiovascular risk factors (diabetes, hypertension or dyslipidaemia), as well as variable evolution time and treatment objectives (primary or secondary prevention of CVD) may allow us to identify in which studies the legacy effect may be more or less relevant. However, some limitations should be taken into account. First, loss of patients during post-interventional follow-up can reach 25–30% of subjects included in the trial (attrition bias), therefore data collection during this period may not have been as exhaustive as in the intervention period. The publication of only those trials with positive results (publication bias) and the absence of blindness in several trials (co-intervention bias) should also be considered. Furthermore, the inclusion of post hoc analysis together with the detection of the legacy effect is influenced by different confounding factors (recommended therapeutic targets, adherence to treatment, global cardiovascular risk), which may lead to a questionable interpretation of the results. Finally, another limitation that must be taken into account is the fact that few non-pharmacological studies (i.e., dietary intervention studies) have been carried out. Although dietary recommendations are included in most studies, they are usually quite generic, and it is also difficult to know the degree of adherence to them.

In conclusion, there is sufficient data to suggest the existence of a legacy effect after intensive intervention on cardiovascular risk factors in subjects with moderate-high vascular risk. However, this effect is not equivalent for all risk factors and could be influenced by patient characteristics, disease duration and the type of intervention performed. Currently, the available evidence suggests that the legacy effect would be greater in subjects with moderate-high cardiovascular risk but without known CVD, especially in patients with recent-onset diabetes. However, we should not withdraw any treatment to prevent CVD in these individuals as the level of available evidence on the legacy effect is low to moderate. Further investigation should be promoted to determine whether there is a legacy effect associated with nutritional interventions.

## Figures and Tables

**Table 1:** Studies assessing the legacy effect after intensive glycaemic control.

	Glycaemic Control				
	DCCT/EDIC (*n* = 1.441/1.394) *	UKPDS (*n* = 4.209/3.277) *	ACCORD (*n* = 10.251/8.601) *	VADT (*n* = 1.791/1.655) *	ADDITION (*n* = 153,107 Screened/125,083 Unscreened) *
**In-trial follow-up duration/total follow-up duration**	6.5 years/17 years	10 years/20 years	3.7 years/8.8 years	5.6 years/15 years	5.3 years/10 years
**Patient characteristics**	Mean age 27 years; T1DM	Mean age 56.4 years; newly diagnosed T2DM	Mean age 62.2 years; long-standing duration T2DM (10 years)	Military veterans; mean age 60.4 years; poorly controlled and long duration T2DM (11.5 years)	Mean age 59.9 years; newly diagnosed T2DM after regular screening
**Type of intervention**	Intensive vs. standard therapy (glycaemic target 70–120 mg/dL vs. no goals)	Intensive vs. standard therapy (sulfonylurea/insulin/metformin vs. DR)	Intensive vs. standard therapy (HbA1c target <6% vs. <7.9%)	Intensive vs. standard glucose control (HbA1c targets <6% vs. <9%)	Intensive vs. routine diabetes care (HbA1c target <7%)
**Dietary intervention**	Standard care	3-month diet in both groups. DR in control group	Standard care	Standard care	Standard care
**In-trial outcomes**	Reduction in retinopathy, neuropathy and development of microalbuminuria in the intensive treatment group	Reduction in microvascular complications, MI and total mortality in the intensive-therapy group	Reduction in nonfatal MI and increased cardiovascular and total mortality in the intensive-therapy group	Significant reduction in progression to microalbuminuria in the intensive-therapy group	Reduction in fatal and nonfatal CVD
**Legacy effect**	Yes	Yes	No	No	Yes
**Effects in the post-trial follow-up**	Reduction in the risk of nephropathy, nonfatal MI, stroke and cardiovascular death with intensive treatment	Reduction in microvascular disease (maintained in the sulfonylurea-insulin group), MI and death from any cause in the intensive-therapy group	A trend to reduction in nonfatal MI, nonfatal stroke and death from any cause, and persisted increase in death from cardiovascular causes in the intensive-therapy group	Reduction in cardiovascular events and a trend to reduction in cardiovascular and total mortality in the intensive-therapy group after 10 years of follow-up	Significantly lower risk in all-cause mortality and cardiovascular events

Abbreviations: CVD: cardiovascular disease; DR: dietary restriction; HbA1c: glycated haemoglobin; MI: myocardial infarction; T1DM: type 1 diabetes mellitus; T2DM: type 2 diabetes mellitus; DCCT: Diabetes Control and Complications Trial; EDIC: Epidemiology of Diabetes Interventions and Complications; UKPDS: United Kingdom Prospective Diabetes Study; ACCORD: Action to Control Cardiovascular Risk in Diabetes; VADT: Veterans Affairs Diabetes Trial; ADDITION: Anglo-Danish-Dutch study of Intensive Treatment in People with Screen-Detected Diabetes in Primary Care. * The number of randomised patients enrolled in the interventional phase and the cohort of post-trial follow-up, respectively.

**Table 2:** Studies assessing the legacy effect after intensive BP control.

	Blood Pressure Control					
	HDS (UKPDS) (*n* = 1.148/884) *	SHEP (*n* = 4.736/1.885) *	ALLHAT (*n* = 32.804/27.755) *	ROADMAP (*n* = 4.447/1.758) *	ANBP2 (*n* = 6.083/5.378) *	ASCOT (*n* = 8.580/7.302) *
**In-trial follow-up duration/Total follow-up duration**	4 years/10 years	4.5 years/22 years	4.9 years/8–13 years	3.2 years/6.5 years	4.1 years/10 years	5.5 years/16 years
**Patients characteristics**	Mean age 56.4 years; newly diagnosed T2DM and hypertension	Mean age 71.6 years; isolated systolic hypertension (≥160 mm Hg) with no pre-existing CVD	Mean age 66.9 years; ≥55 years, hypertensive and at least one additional risk factor for CHD events	Mean age 61.2 years; T2DM with cardiovascular risk factors and normoalbuminuria	Mean age 71.8 years; average systolic BP of 160 mm Hg or average diastolic BP of 90 mm Hg, without recent cardiovascular events	Mean age 64 years; hypertension with at least three other cardiovascular risk factors without CHD
**Type of intervention**	Tight vs. less tight BP control with captopril or atenolol (target <150/85 vs. <180/105 mm Hg)	Chlortalidone vs. placebo	Amlodipine, lisinopril or doxazosin vs. chlortalidone	Olmesartan vs. placebo	ACE inhibitor vs. diuretic therapy	Amlodipine-based regimen vs. atenolol-based regimen
**Dietary intervention**	Standard care	Standard care	Standard care	Standard care	Standard care	Standard care
**In-trial outcomes**	Reduction in MI, sudden death and microvascular complications	Reduction in fatal or nonfatal strokes, MI and heart failure	No differences in CHD or nonfatal MI	Increased time to the onset of microalbuminuria	Less risk of cardiovascular and all-cause mortality in control group	Reduction in cardiovascular events and mortality in amlodipine-based group
**Legacy effect**	No	Yes	No	Yes	No	Yes
**Effects in the post-trial follow-up**	Reduction in peripheral vascular disease in the tight BP control	Approximately one day gained in life expectancy free from cardiovascular death for each month of active therapy in the chlortalidone group	Higher hospitalised and fatal heart failure on amlodipine group and higher stroke mortality on lisinopril group	Significant reduction in CHF, retinopathy and non-significant reduction in the onset of microalbuminuria in the olmesartan group	No differences in cardiovascular outcomes or all-cause mortality	Significant fewer stroke deaths on amlodipine-based group

Abbreviations: ACE: angiotensin-converting enzyme; BP: blood pressure; CHD: coronary heart disease; CHF: congestive heart failure; CVD: cardiovascular disease; MI: myocardial infarction; T2DM: type 2 diabetes mellitus; HDS: Hypertension in Diabetes Study; UKPDS: United Kingdom Prospective Diabetes Study; SHEP: Systolic Hypertension in the Elderly Program; ALLHAT: Antihypertensive and Lipid Lowering Treatment to Prevent Heart Attack Trial; ROADMAP: Randomised Olmesartan And Diabetes MicroAlbuminuria Prevention; ANBP2: Second Australian National BP study; ASCOT: Anglo-Scandinavian Cardiovascular Outcomes Trial. * The number of randomised patients enrolled in the interventional phase and the cohort of post-trial follow-up, respectively.

**Table 3:** Studies assessing the legacy effect after lipid-lowering intervention.

	Lipid-Lowering Intervention			
	ALLHAT-LLT (*n* = 10.355/1.672) *	WOSCOPS (*n* = 6.596/6.408) *	ASCOT-LLA (*n* = 4.605/4.432) *	ACCORD-Lipid (*n* = 940/765) *
**In-trial follow-up duration/Total follow-up duration**	4.9 years/8–13 years	4.9 years/20 years	3.3 years/15.7 years	5 years/9.7 years
**Patients characteristics**	Men and women; mean age 66.4 years; hypertensive and at least one additional risk factor for CHD events	Men; mean age 55 years; high LDL cholesterol without CHD	Men and women; mean age 64 years; hypertension with at least three additional cardiovascular risk factors and total cholesterol ≤6.5 mmol/L	Men and women; mean age 61.4 years; long-standing T2DM with LDL between 60–180 mg/dL, HDL-C <55 mg/dL and triglycerides <750 mg/dL or <400 mg/dL on treatment
**Type of intervention**	Pravastatin vs. usual treatment	Pravastatin vs. placebo	Atorvastatin vs. placebo	Simvastatin + fenofibrate vs. simvastatin + placebo
**Dietary intervention**	Standard care	Standard care	Standard care	Standard care
**In trial outcomes**	No differences in all-cause mortality	Reduction in all-cause mortality, and death or hospitalisation for CHD	Reduction in nonfatal MI and fatal CHD	No benefits in cardiovascular outcomes and mortality; less major CHD events in hyperlipidaemic patients
**Legacy effect**	No	Yes	Yes	Yes
**Effects in the post-trial follow-up**	No reduction in CHD events and cardiovascular mortality	Reduction in all-cause and cardiovascular mortality, and lower cumulative hospitalisation event rates for any coronary event, MI, and heart failure in the pravastatin group	Reduction in cardiovascular mortality in the atorvastatin group	Reduction in all-cause and cardiovascular mortality, nonfatal MI, CHF and major CHD in the fenofibrate group; reduction in all-cause mortality in combined statin-fibrate arm

Abbreviations: CHD: coronary heart disease; CHF: congestive heart failure; HDL: high-density lipoprotein; LDL: low-density lipoprotein; MI: myocardial infarction; T2DM: type 2 diabetes mellitus. LLT: Lipid-lowering treatment; WOSCOPS: West of Scotland Coronary Prevention Study; LLA: lipid-lowering arm. * The number of randomised patients enrolled in the interventional phase and the cohort of post-trial follow-up, respectively.

**Table 4:** Studies assessing the legacy effect after multifactorial intervention.

	Multifactorial Intervention	
	ADVANCE (*n* = 11.140/8.494) *	Steno-2 (*n* = 160/96) *
**In-trial follow-up duration/Total follow-up duration**	4.5 years (BP control) and 5-years (glycaemic control)/10 years	7.8 years/21.2 years
**Patient characteristics**	Men and women; mean age 66 years; T2DM with at least one additional risk factor for CVD and aged ≥55 years	Men and women; mean age 55.1 years; T2DM and microalbuminuria
**Type of intervention**	Combination of perindopril-indapamide vs. placebo. Intensive (gliclazide) vs. standard glucose control	Intensified multifactorial intervention (behavioural and pharmacological approaches) vs. standard therapy
**Dietary intervention**	Standard care	Dietary advice (total daily intake of fat ≤30% of total daily energy intake and less of 10% as saturated fat)
**In-trial outcomes**	Reduction in total and cardiovascular mortality in the intensive BP-lowering group. Reduction in nephropathy in the intensive-glycaemic control group	Reduction in the risk for progression to nephropathy, retinopathy and autonomic neuropathy, and decreased risk in cardiovascular complications and all-cause mortality combined in the intensive treatment group
**Legacy effect**	Yes (BP control)/No (glycaemic control)	Yes
**Effects in the post-trial follow-up**	Reduction in all-cause and cardiovascular mortality in the intensive BP-lowering group	Reduction in all-cause and cardiovascular mortality, and microvascular complications (nephropathy, retinopathy and autonomic neuropathy) in the intensive-therapy group

Abbreviations: BP: blood pressure; CVD: cardiovascular disease; T2DM: type 2 diabetes mellitus. * The number of randomised patients enrolled in the interventional phase and the cohort of post-trial follow-up, respectively.
